# The role of hepatic fat accumulation in pathogenesis of non-alcoholic fatty liver disease (NAFLD)

**DOI:** 10.1186/1476-511X-9-42

**Published:** 2010-04-28

**Authors:** Qing Liu, Stig Bengmark, Shen Qu

**Affiliations:** 1 Deaprtment of Endocrinology, Tenth People's Hospital, Tongji University, Shanghai 200072, China; 2 Departments of Hepatology and Surgery, University College London, 69 - 75 Chenies Mews, London, WC1E 6HX, UK; 3 Beijing You An Hospital, Beijing 100006, China

## Abstract

Nonalcoholic fatty liver disease is increasingly regarded as a hepatic manifestation of metabolic syndrome, and the severity of nonalcoholic fatty liver disease seems to increase in parallel with other features of metabolic syndrome. Excess lipid accumulation in the liver cells is not only a mediator of Metabolic Syndrome and indicator of a lipid overload but also accompanied by a range of histological alterations varying from 'simple' steatosis to nonalcoholic steatohepatitis, with time progressing to manifest cirrhosis. Hepatocellular carcinoma may also occur in nonalcoholic steatohepatitis -related cirrhosis with a mortality rate similar to or worse than for cirrhosis associated with hepatitis C. This review summarizes the knowledge about the causal relationship between hepatic fat accumulation, insulin resistance, liver damage and the etiological role of hepatic fat accumulation in pathogenesis of extra- and intra-hepatic manifestations. Special emphasis is given suggestions of new targets treatment and prevention of nonalcoholic fatty liver disease.

## Introduction

Nonalcoholic fatty liver disease (NAFLD), no more than 15 years ago considered as rare, has now reached epidemic propertions in China and has a major clinical and research priority [1]. It is only since a decade or so that nonalcoholic fatty liver disease is considered to be a manifestations of insulin resistance[2,3]. The fact that insulin resistance and compensatory hyperinsulinaemia have central etiologic roles in the development of both metabolic syndrome and NAFLD makes the excessive accumulation of triglycerides (TG) in the liver especially interesing [4]. The severity of NAFLD increases in parallel with other features of the metabolic syndrome, supporting that NAFLD is its hepatic manifestation [5].

Although current therapy presently is limited to suggestions of lifestyle changes and control of associated metabolic disorders, experimental studies and clinical observations do suggest also other modalities of treatment. As an example, a proof-of-concept study suggest that administration of pioglitazone improvements in both metabolic and histological manifestations of disease in subjects with nonalcoholic steatohepatitis[6].

NAFLD has the potential to progress through the inflammatory phase of nonalcoholic steatohepatitis (NASH) to fibrosis, cirrhosis (20%), and in some cases (9%) to liver failure or hepatocellular carcinoma (HCC) (1%) [7,8]. The reasons why some patients develop advanced disease while others have non- och less progressive liver disease are not fully understood. The mechanism of liver injury in NAFLD/NASH is suggested to be a 'two hit phenomenon' where the 'first hit' consists in inflammation and development of steatosis, which sensitizes the liver to a variety of 'second hits' lead to fibrosis [7,9]. It is also increasingly recognized that fat-induced insulin resistance in the liver causes activation of proinflammatory pathways, which will lead to modification of this 'two hit' process.

Identifying patients at risk for transition from hepatic steatosis to NASH and fibrosis with noninvasive techniques are the great challenges in hepatology [9]. Therefore, combining of the hepatologist's knowledge of human hepatic pathology and pathophysiology with the endocrinologist's knowledge of insulin signaling and the regulation of glucose and lipid metabolism in the liver will help to better understand the process, and hopefully also, help to find new targets for the treatment and prevention of NAFLD.

### The causal relationship between hepatic fat accumulation and insulin resistance

**Figure 1:**
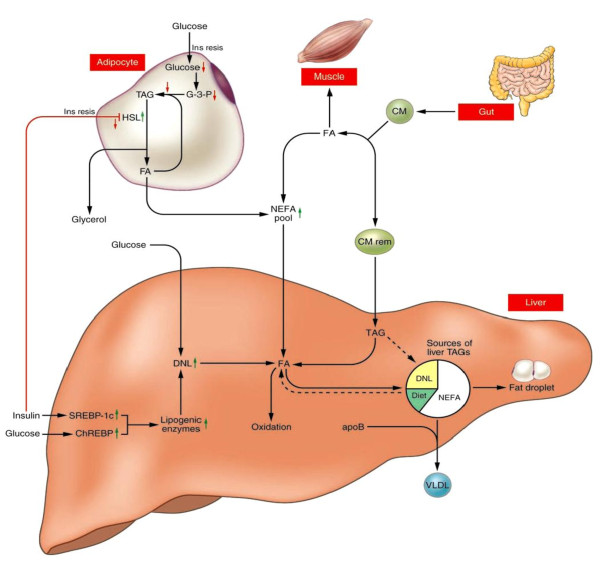
**Sources of fatty acids stored in liver and secreted via lipoproteins in patients with nonalcoholic fatty liver disease (Kerry L. Donnelly, et al, J. Clin. Invest. 2005;115:1343-1351)**.

Hepatocellular steatosis is the hallmark of NAFLD. The histological criterion for the diagnosis of NAFLD is the presence of fat in more than 5% of the hepatocytes. The accumulation of fat usually starts in zone 3 and will in more severe cases may occupy whole acinus[10-12]. The lipid droplets in liver consist mainly in triglycerides. The contribution of different non-esterified fatty acids (NEFA) sources to hepatic triglycerides parallel to the VLDL triglycerides, includes: circulating NEFA pool contributed 81% of hepatic and VLDL triglycerides in fasting conditions and 61% postprandially, dietary NEFA pool contributed 10% in fasting conditions and 26% postprandially, whereas de novo fatty acid(DNL) contributed 7% in fasting and 9% postprandially[13](Fig 1).

Insulin resistance is defined as an inadequate response to the physiologic effects of circulating insulin in specific target tissues, such as skeletal muscle, liver, and adipose tissue,. The subjects with impaired glucose tolerance (IGT) demonstrate a marked muscle insulin resistance but only mild hepatic insulin resistance. Subjects with impaired fasting glucose (IFG), however, demonstrate severe hepatic insulin resistance but normal or near-normal muscle insulin sensitivity[14]. Epidemiological studies demonstrate that the two categories constitute two distinct populations, which only partially overlap, suggesting that IGT and IFG represent two distinctly different metabolic abnormalities [15]. However, it must be regarded as undisputable, that a strong association exists between insulin resistance and excess lipid accumulation in nonadipose tissues, particularly muscle and liver.

Although an association between NAFLD and insulin resistance is well accepted, it remains unclear whether insulin resistance causes the excessive accumulation of TG in liver, or it is the increase in TG itself or of its metabolite intermediates that plays the most causal role in the development of hepatic or systemic insulin resistence [16]. Some investigators postulate that liver fat accumulation is a result, rather than cause, of peripheral insulin resistance in obesity. In skeletal muscle, peripheral IR will primarily affect a large portion of the total glucose uptake (>80-90%), while in adipose tissue, insulin resistance will induce an impaired anti-lipolytic action of insulin and increased release of NEFA [17,18]. Elevated plasma concentrations of glucose and fatty acids may promote hepatic fatty acid and triglyceride uptake and synthesis and impair β-oxidation [19]. As a result, an accumulation of triglycerides in the liver will occurs, leading to hepatic steatosis. The proposed mechanism for insulin resistance leading to diabetes is thus: IR in skeletal muscle → peripheral and portal vein hyperinsulinemia → hepatic steatosis → hepatic IR → DM2.

However, several observations indicate that steatosis can be caused also by intra-hepatic alterations in glucose and fat metabolism, which are independent of extrahepatic conditions[20,21]. Three consecutive days of feeding a high fat diet is known, also in the absence of significant peripheral fat accumulation to cause hepatic fat accumulation and hepatic insulin resistance and this prior to the development of peripheral insulin resistence[22]. This study demonstrated that short term fat feeding will cause a 3-fold increase in liver and fatty acyl-CoA content, but not alter fasting plasma glucose concentration and the basal rate of endogenous glucose production (EGP), indicating that hepatic fat accumulation from dietary NEFA pool is not necessarily associated with insulin resistance[23].

Decreased insulin clearance has also been suggested as another pathway for hyperinsulinemia in liver. Insulin clearance is decreased in advanced liver disease, which is regarded as one of the main causes of hyperinsulinemia in iver cirrhosis [24]. The first organ to be impacted following insulin secretion is the liver, where a substantial proportion of secreted insulin is cleared via receptor mediated process [25,26], estimated in dogs to be app 50% [27,28] and in humans 40-80% [25,29-32]. Both hepatic insulin sensitivity and whole body insulin clearance were in this study measured directly by the euglycemic hyperinsulinemic insulin clamp technique and liver fat measured by proton magnetic resonance spectroscopy in a group of nondiabetic subjects. The content of liver fat correlated significantly with impaired clearance of insulin (*r *= 0.43, *P *< 0.0001) and with hepatic insulin sensitivity (*r *= -0.40, *P *= 0.0002) [33,34]. The extent to which hepatic fat accumulation and impaired insulin clearance contribute to hyperinsulinemia can not yet be regarded as fully understood.

**Figure 2:**
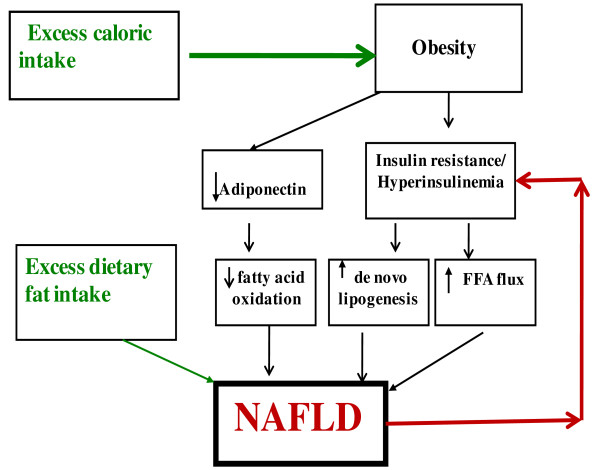
**Diagram of potential sources of and mechanisms for the accumulation of fat in the liver**. Excess caloric intake plays a central role for inducing obesity and thus contributing to insulin resistance. Obesity may additionally impact fat accumulation in the liver by decreasing adiponectin levels and insulin resistance contributes to NAFLD directly by increasing *de novo *lipogenesis and indirectly by increasing FFA flux to the liver via decreased inhibition of lipolysis. In addition, excess dietary fat intake directly could also contribute to a causal role in hepatic steatosis(modified from **Kristina M**. The Journal of Clinical Endocrinology & Metabolism 2006; 91: 4753-4761).

As discussed above (Fig 2), the peripheral insulin resistance could well contribute to a causal role for hepatic steatosis, but NAFLD and hepatic insulin resistance is also regarded as a primary cause of of T2 MD.

The symptoms of NAFLD vary considerably from person to person, as some individuals may have few or no noticeable symptoms until they are incidentally diagnosed by elevated levels of aminotransferase and by ultrasound. Other individuals will present with extremely high levels of hyperglycemia and classical symptoms of diabetes such as thirst, frequent urination, and weight loss.

### The accumulation of triglycerides in liver is not equal to increased liver damage

Minimal histopathology criteria are used for diagnosis of NAFLD. Assessment of steatosis with reproducible semiquantitative methods use a three-stage scale to evaluate the percentage of liver parenchyma occupied by steatotic hepatocytes: I. 0-33% (or 0-5%, 5-33%), II 33-66%and III more than 66%[12]. Although hepatic steatosis is a key component of steatohepatitis, it is not known if patients with greater amounts of steatosis are more likely to develop steatohepatitis than those with mild steatosis. The histological definition of steatohepatitis does not require a particular amount of steatosis, and, steatosis will often decrease and may even be completely absent in cases with advanced fibrosis and cirrhosis [11,35].

Several findings suggest that hepatic triglycerides per se are not toxic and may, in fact, even protect the liver from lipotoxicity by buffering the accumulation of fatty acid[36,37]. Yamaguchi et al [38] demonstrated that inhibition of TG synthesis (mediated through the reduction in DGAT2 activity) will improve liver steatosis, but at a price of increased liver damage. Levels of hepatic fatty acids, cytochrome P450, and markers of lipid peroxidation and oxidant stress, as well as fibrosis, were in this study all markedly increased, Recent findings confirm that the ability to synthesize triglycerides may, in fact, be protective in obesity. When hepatocyte triglyceride synthesis is inhibited, free fatty acids will accumulate in the liver, and swtimulate oxidizing systems that increase hepatic oxidative stress and liver damage[39,40]. Furthermore, feeding an n-3 PUFA-enriched diet failed to prevent lipotoxic hepatocellular injury and inflammatory recruitment, although activated PPAR alpha and suppressed hepatic de novo lipogenesis[39].

The differential toxicity of various FAs is relate directly to its ability to promote triglyceride accumulation[39]. The exogenous or endogenous generated unsaturated FAs will reduce palmitate-induced apoptosis by promoting palmitate incorporation into triglycerides. Moreover, oleic acid, as well as palmitic acid, is toxic to cells with impaired capacity to synthesize triglycerides. Exposure of human hepatocytes to monounsaturated fatty acids (MUFAs) results in lipid accumulation without any changes in cell viability. In contrast, cells incubated with saturated fatty acids (SFAs) decrease significantly their viability in addition yo increasing their caspase activation and degree of apoptosis, parallel to only minor lipid droplet accumulation[39].

Stearoyl-CoA desaturase-1 (SCD-1), the enzyme that converts SFA to MUFA, appears to provide an important metabolic control mechanism in the biochemistry and physiology of lipid metabolism. Genetic or pharmacological inhibition of SCD1, sensitizes hepatic cells to SFA-induced apoptosis. SCD1-/- mice on the MCD diet demonstrate decreased steatosis and markedly increased hepatocellular apoptosis, liver injury, and fibrosis compared to SCD1+/+ mice, whereas feeding MUFA prevents the MCD-induced injury[41].

The studies referred to above clearly show that increased serum free fatty acid, rather than the excess lipid accumulation in liver cells serves as a parameter of degree of liver damage, and might be considered as a future target for preventing and therapy of steatohepatitis in patients with NAFLD. Findings that inhibition of the expression of SCD-1 might also be beneficial in the treatment of obesity, diabetes, and other metabolic diseases [42-45].

However, it is still quite possible that under the conditions of enhanced TG synthesis, the capacity will be insufficient to buffer detoxifying of excess fatty acids. Liver damage could then occur as a result of fatty acid-induced lipotoxicity and/or of a combination of both lipotoxicity and severe steatosis. A recent study shows that the severity of steatosis in NAFLD on histopathology is positively associated with lobular inflammation, zone 3 fibrosis and definite steatohepatitis, suggesting that NAFLD patients with severe steatosis (affecting >66% of liver parenchyma) are most likely to have NASH [46]. We currently mainly relying on liver biopsies to confirm the diagnosis and indicate prognosis, as this far hepatic ultrasound and serum transaminases are of limited value in estimation of hepatic inflammation and fibrosis, However, ultrasonography is still by far the most common method of diagnosing NAFLD in clinical practice, especially as it has a very good sensitivity and specificity in detecting severe steatosis(>33% fat on liver biopsy), although the sensitivity of ultrasonography is not satisfactory, particularly when hepatic fat infiltration is above 33% [47,48]. The positive relationship between the severity of steatosis and other elements of NASH (i.e. inflammation, ballooning, and fibrosis) provides noninvasive techniques to identify patients at risk for transition from hepatic steatosis to NASH and fibrosis. However, we are still unable to determine if patients with severe steatosis do faster progress to full blown steatohepatitis than those with milder forms of steatosis.

### Fat-induced activation of chronic SIRS and NASH in the progression of NAFLD

NAFLD is a liver disease with a wide histological spectrum ranging from 'simple' steatosis with a generally benign course to nonalcoholic steatohepatitis (NASH), which usually is a more progressive form of the disease [11]. Progressive fibrosis occurs in 10-15% of patients with NASH and cirrhosis may develop in 15-25% of the cases [49,50]. Once developed, 30% to 40% of these patients succumb a liver-related death over a 10-year period [51,52], a mortality rate similar to[53,54] or worse than cirrhosis associated with hepatitis C. NASH-associated cirrhosis can also develop into into a subacute liver failure, or progress to hepatocellular cancer [55], as well as reoccur after liver transplantation.

The reasons why some patients develop advanced disease, while others have indolent liver disease, are not fully understood. The mechanism of liver injury in NAFLD/NASH is thought to follow, as mentioned above, a 'two hit pathway during which the steatosis sensitizes the liver to a variety of second hits, which lead to fibrosis and inflammation[7]. The 'first hit', hepatic triglyceride accumulation, or steatosis, increases the susceptibility of the liver to injury- mediated 'second hits'-changes, such as inflammatory cytokines/adipokines, mitochondrial dysfunction and oxidative stress, which in turn promote the development to steatohepatitis and/or fibrosis [9,56]. There is an increasing recognition of the fact that fat-induced insulin resistance causes activation of proinflammatory pathways in the liver, which with time might lead to modification of the theory.

Most studies on the role of hepatic innate immunity in NAFLD has been generated by studying leptin-deficient (ob/ob) mice and leptin-resistant (fa/fa) rats, animals, which spontaneously develop fatty livers and naturally exhibit features, such as insulin resistance, obesity, and dyslipidemia, which in its turn in humans are strongly correlated with NAFLD [57-60]. It is increasingly documented that obesity without simultaneous inflammation does not result in peripheral insulin resistance, and similarly, the steatosis will not lead to liver inflammation (NASH) and/or hepatic insulin resistance [57]. Wild type mice fed a high-fat diet, will normally develop obesity with simultaneous inflammation, insulin resistance and mild Type 2 diabetes. On the contrast, chimerical mice, lacking immune cells will become obese, but have a striking absence of insulin resistance. In addition, their livers will remain normal [61-63]. Among the proinflammatory molecules, TNF-α has been proposed to be the key link between obesity and insulin resistance. TNF-α is usually over- expressed in adipose tissues of obese animals and humans, and obese mice lacking TNF-α and/or its receptor demonstrate protection against development of insulin resistance[64,65].

To date, many lines of evidence seem to show that in humans chronic activation of proinflammatory pathways of insulin targetted cells can result in obesity/steatosis -related insulin resistance. The influence of circulating and hepatic levels of proinflammatory cytokines TNF-α, IL-6, and C-reactive protein (CRP) in patients with NAFLD and their correlation with disease severity are well studied. Patients with NASH have generally significantly higher levels of serum TNF-α and IL-6 than seen in patients with simple steatosis. NASH is, when compared to simple steatosis, associated with higher levels of soluble TNF receptor 1 (sTNFR1) and soluble IL-6 receptor (sIL-6R) [66,67]. In addition, the expression is higher in those patients with more severe NASH. The findings that inflammation and insulin resistance often seem to occur in parallel and to reinforce each other via positive feedback mechanisms, suggesting that disruption of the interface between inflammatory and metabolic pathways is central in the pathogenesis and progression (second hit) of NAFLD and also increase the risk of development of insulin resistance (Type 2 diabetes). Given the role of TNF-α in inducing both necroinflammation and IR, anti-TNF agents have been considered for treatment of NASH. Reducing TNF-α signaling either by TNF-α knocking out mechanisms or by infusion of blocking antibodies reduces insulin resistance in obese rodents [68]. Pentoxifylline, a TNF-α inhibitor, has also been used for the same purpose in patients with NASH. Two smaller studies in NASH patients report significant improvements in AST and ALT and also significant improvement in insulin resistant after pentoxyfylline treatment [69,70], supporting the hypothesis that anti-inflammatory treatments have favorable effects on NASH. However, TNF-α antibody treatment will not alter insulin sensitivity in humans [71]. Attempts in this study to over a period of 4 weeks neutralize TNF-alpha with an engineered human anti-TNF-alpha antibody (CDP571) had no effect on insulin sensitivity in obese non-insulin-dependent diabetes subjects with rheumatic diseases.

Fat can cause insulin resistance by prompting the activation of select serine kinases within a variety of insulin sensitive cells. The three serine kinases most strongly implicated in the pathogenesis of fat-induced insulin resistance are Jun N-terminal kinase (JNK), inhibitor of nuclear factor κB (NF-κB) kinase(IKK), and novel isoforms of protein kinase C (PKC) [72-74]. These enzymes will, alone or in combination, phosphorylate regulatory serine residues on the insulin receptor substrates IRS-1 and IRS-2, leading to the downmodulation of normal insulin-stimulated tyrosine phosphorylation and interfering with physiologic insulin [57]. Among these, JNK and IKK are significantly effective proinflammatory signaling molecules. IKK and JNK cause inflammation by promoting formation of the transcription factors AP-1 and NF-κB, which activate transcription of a multitude of proinflammatory genes, cytokines, chemokines, and cell adhesion molecules[75]. A causal connection between fat-related activation of serine kinases and insulin resistance has been demonstrated in adipose tissue [76] and muscle [77] as well as liver [78-81]. Mice heterozygous for IKKβ are protected against the negative effects of a high-fat diet-associated obesity as well as development of insulin resistance [82]. Silencing of IKKβ will also prevent TNF-alpha-induced impairments in the action of insulin on Akt phosphorylation and glucose uptake in primary human skeletal muscle myotubes from non-diabetic subjects [83,84].

**Figure 3:**
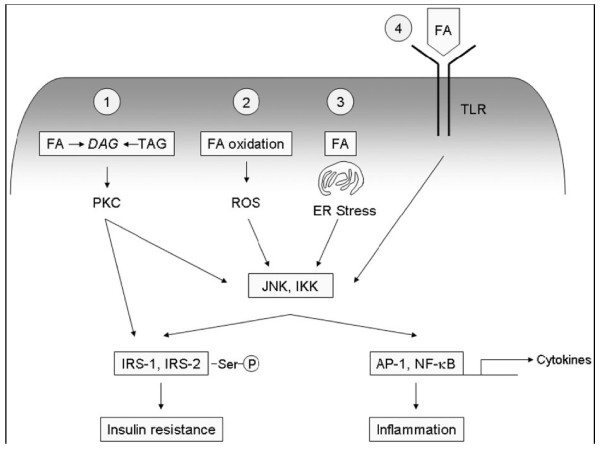
**Activation of inflammatory signaling pathways by fat(Hepatology. 2008;48:670-8)**.

Also, fatty acids and high-fat feeding can, even in the absence of obesity or systemic insulin resistance, directly induce inflammatory signaling through mammalian toll-like receptors (TLRs) in the liver [85,86]. TLRs are cellular pattern-recognizing receptors (PRRs) that recognize the molecular patterns of pathogens, such as Gram-negative endotoxin. After engaging the pathogenic patterned ligands *via *a receptor-driven signaling cascade do they activate the transcription factor NF-κB, resulting in increased expression of proinflammatory cytokines, which in its turn trigger inflammation. Such rapid, innate cellular responses serve as the first line of host defense against infection by pathogens. Increasing evidence suggests that TLRs also recognize host-derived ligands and interact with these endogenous ligands, generating tissue injury and cell death [87]. Thus TLRs will act as PRRs that paralle with the lipotoxicity and stress of endoplasmic reticulumt, use signaling mechanisms to solicit inflammation and fat-induced insulin resistance (Fig 3). Such efforts should also reveal a wealth of potential targets for interventions and drug development that prevent NASH[88]. Probiotics, which reportedly suppress TLR-related responses by altering the intestinal flora [89,90], reduce inflammation and improve the liver in animals and humans with fatty livers[91,92]. Similarly, dietary n-3 polyunsaturated fatty acids, which are known inhibitors of TLR signaling[93], will have the capacity to suppress necro-inflammation and fibrosis in experimental fatty liver disease[94].

**Figure 4:**
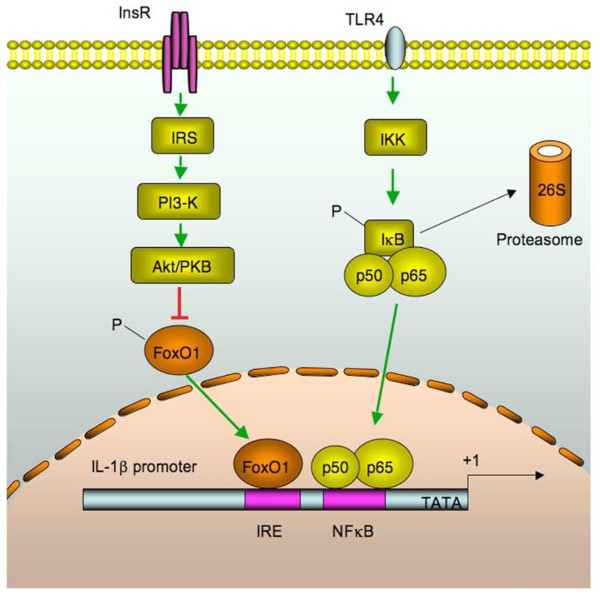
**Convergence of the FoxO1 and NFκB pathways in IL-1β gene expression: FoxO1 targets at the IRE DNA motif of the IL-1β promoter for trans-activation**. This effect along with the activation of the NFκB pathways synergistically promotes macrophage production of proinflammatory cytokine IL-1β. (Dongming Su et al. FoxO1 Links Insulin Resistance to Proinflammatory Cytokine IL-1beta Production in Macrophages. in Press).

FoxO1 is a forkhead transcriptional factor that acts to mediate insulin action on target gene expression in peripheral cells [95-97]. More recent studies show that FoxO1 signaling through NF-kB plays a significant role in proinflammatory cytokine production and insulin resistance (Fig 4). FoxO1 stimulate selectively macrophage production of IL-1β, but not IL-6, TNFα, and IFN-γ.

FoxO1 is known to mediate the inhibitory effect of insulin on target gene expression. In the absence of insulin, FoxO1 acts in the nucleus as an enhancer of target gene expression, whereas in the presence of insulin, FoxO1, phosphorylated by Akt/PKB, will contribute to inhibition of target gene expression. The phosphorylation-dependent protein trafficking mechanism is crucial for the regulation of FoxO1 transcriptional activity within cells [98-100]. The IL-1β promoter contains sites for both FoxO1 and NFkB target that are juxtapositioned within the promoter-proximal region of the Il1b gene in mice, rats and humans, implying evolutionally conserved mechanisms or FoxO1 signaling through the NFkB pathway in regulation of proinflammatory cytokines. Amelioration of insulin resistance with blockage of IL-1β signaling via its receptor antagonist is associated with improving diabetes [101].

## Conclusion

The molecular mechanisms underlying NAFLD and treatment options are this far poorly understood. Clearly, numerous factors are associated with increased activation of hepatic innate immune system, which is central to the development of fat-related insulin resistance and also plays an important role in fat-related liver damage (NASH). NASH is seemingly a much multifactorial condition with some pathological end points such as increased hepatic oxidative stress, increased hepatic cytokine expression, direct "lipotoxicity" of excess circulating NEFAs and insulin resistance. Especilly are the serine kinases key factors in the development of insulin resistance within hepatocytes and in other insulin sensitive cells. Especially IKK and JNK, are important components what is described as the two major proinflammatory pathways.

Future exploration of the signaling routes from fat to NF-κB might provide information about hitherto unknown etiological factors behind chronic diseases in addition to those already known. It might also provide information about potentially new targets for drug development and treatment of these diseases.

## Abbreviations

BMI: body mass index; NAFLD: nonalcoholic fatty liver disease; NASH: nonalcoholic steatohepatitis; DNL: *de novo *lipogenesis; NEFA: non-esterified fatty acids; LDL: very low density lipoproteins; TG: triglycerides; CVD: cardiovascular disease; IFG: impaired fasting glucose; IGT: impaired glucose tolerance; SCD1: stearoyl-CoA desaturase-1; IR: insulin resistance; DM2: diabetes Mellitus Type 2; CRP: C-reactive protein; IL-6: interleukin-6; sTNFR1: TNF receptor 1; sIL-6R: soluble IL-6 receptor; TLRs: Toll-like receptors; RAGE: receptor for advanced glycation end products; SIRS: systemic inflammatory response syndrome; JNK: Jun N-terminal kinase; NF-κB: nuclear factor κB; IKK: IκB kinase; PKC: protein kinase C.

## Competing interests

The authors declare that they have no competing interests.

## Authors' contributions

QL, SB and SQ conceived the study, its design and drafted the manuscript. All authors read and approved the final manuscript.
